# MycoCAP - *Mycobacterium* Comparative Analysis Platform

**DOI:** 10.1038/srep18227

**Published:** 2015-12-15

**Authors:** Siew Woh Choo, Mia Yang Ang, Avirup Dutta, Shi Yang Tan, Cheuk Chuen Siow, Hamed Heydari, Naresh V. R. Mutha, Wei Yee Wee, Guat Jah Wong

**Affiliations:** 1 Genome Informatics Research Laboratory, High Impact Research Building, University of Malaya, 50603 Kuala Lumpur, Malaysia; 2 Department of Oral Biology and Biomedical Sciences, Faculty of Dentistry, University of Malaya, 50603 Kuala Lumpur, Malaysia; 3 Department of Molecular Genetics, University of Toronto, ON M5S3E, Canada

## Abstract

*Mycobacterium* spp. are renowned for being the causative agent of diseases like leprosy, Buruli ulcer and tuberculosis in human beings. With more and more mycobacterial genomes being sequenced, any knowledge generated from comparative genomic analysis would provide better insights into the biology, evolution, phylogeny and pathogenicity of this genus, thus helping in better management of diseases caused by *Mycobacterium* spp.With this motivation, we constructed MycoCAP, a new comparative analysis platform dedicated to the important genus *Mycobacterium*. This platform currently provides information of 2108 genome sequences of at least 55 *Mycobacterium* spp. A number of intuitive web-based tools have been integrated in MycoCAP particularly for comparative analysis including the PGC tool for comparison between two genomes, PathoProT for comparing the virulence genes among the *Mycobacterium* strains and the SuperClassification tool for the phylogenic classification of the *Mycobacterium* strains and a specialized classification system for strains of *Mycobacterium abscessus*. We hope the broad range of functions and easy-to-use tools provided in MycoCAP makes it an invaluable analysis platform to speed up the research discovery on mycobacteria for researchers. Database URL: http://mycobacterium.um.edu.my

The genus *Mycobacterium* belonging to the family Mycobacteriaceae consists of Gram positive, aerobic, non-motile, non-spore-forming, slightly curved rods^1^. This genus has gained widespread attention due to the fact that it is the causative agent of diseases like leprosy, Buruli ulcer and tuberculosis (TB) in human beings. As per the Global Tuberculosis Report 2014, TB ranks as the second leading cause of death from a single infectious agent with 1.5 million deaths in 2013. Although the mortality rate of TB has come down by 45% since 1990, an estimated 480,000 people developed multidrug-resistant TB (MDR-TB), with an estimated 210,000 deaths from MDR-TB globally in 2013. To make things worse, at an average, an estimated 9% of people with MDR-TB have extensively drug-resistant TB (XDR-TB) as indicated from data reported by 100 countries in 2013^2^,^3^. Presently the mycobacterial species causing tuberculosis are grouped as the *Mycobacterium tuberculosis complex* (MTC)^4^,^5^. Apart from the MTC, the mycobacterial species isolated from environmental sources have also been associated with different diseases in both humans and animals^6^. These pathogenic environmental mycobacteria were called atypical environmental or opportunistic mycobacteria in the past, and now they are designated as mycobacteria other than typical tubercle (MOTT)^7^. One group of such pathogenic environmental mycobacteria is the *Mycobacterium avium* Complex (MAC) causing infections in immunocompromised people, especially with HIV and AIDS.

With the development of the Next Generation Sequencing (NGS) technologies, a large number of mycobacterial genomes are being sequenced and analyzed. It is becoming apparent that the genus is evolving to the changing environment into multidrug-resistant or even extensively drug-resistant strains. The large amount of available genome sequences allows comparative analysis which may give researchers further insights into mycobacteria, particularly the biology, evolution and pathogenicity of the important human pathogens within this genus.

Many genomic databases and analysis platforms have been developed and published for well-studied human pathogens such as PATRIC^8^, but a dedicated comparative analysis platform integrating the mycobacterial genomes and their related information is still missing, despite the availability of their genomics data world-wide. Several online databases, such as the Microbial Genome Database for Comparative Analysis (MBGD)^9^ and also the Integrated Microbial Genomes (IMG) system^10^ provide a wide array of microbial genomes including some *Mycobacterium* strains for comparative genomics analyses. However, these databases may have limited tools for comparative analysis particularly for comparative pathogenomics analyses, which may be important to reveal the cause of infection caused by these pathogens. Moreover, most of the existing biological databases also do not have user-friendly Graphical User Interfaces (GUI), the ability to browse the whole database smoothly or in real time and also the implementation of real-time searching feature.

To accelerate research in mycobacteria, MycoCAP was designed and constructed to provide a specialized *Mycobacterium* genomic encyclopedia and comparative analysis platform, dedicated to the storage and analysis of the rapidly increasing high-throughput genomic data of *Mycobacterium* species. MycoCAP presents all the data in a useful manner that is very easy to access, and enables comparative analyses of these genomic data. MycoCAP provides a comprehensive set of *Mycobacterium* genomic data and a set of useful analysis tools with diverse functionality for data analysis such as PGC for pairwise genome comparison among *Mycobacterium* strains, PathoProT for comparative pathogenomics analysis and SuperClassification, a pipeline for the *Mycobacterium* species and one specifically for the classification of *Mycobacterium abscessus* strains. Other than that, we have also implemented the AJAX-based real-time search feature and MGAB a fast and modern JavaScript-based genome browser customized from JBrowse^11^ into MycoCAP, which facilitates and provides the rapid and seamless searching and browsing experience of the *Mycobacterium* genomes and annotations.

## Results

### Overview of the Web Platform

**Figure 1:**
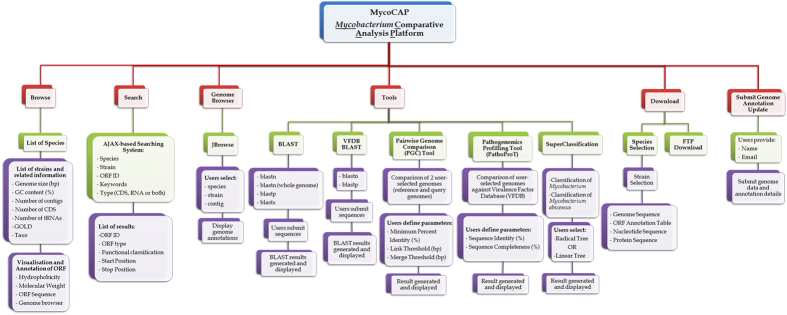
Overview of the functionalities of MycoCAP.

MycoCAP has been designed to be a database for the *Mycobacterium* genus as a whole and houses the genomic data of the *Mycobacterium* species. As more and more mycobacterial genomes are being sequenced, comparative study of multiple *Mycobacterium* genomes could provide us with a detailed view of the relatedness and the variations among the organisms at the genomic level, giving us a better understanding about their physiology. In order to provide useful comparative analysis platform, MycoCAP has also been integrated with new and advanced bioinformatics tools. An overview of the tools and functionalities of MycoCAP is provided in Fig. 1.

**Figure 2:**
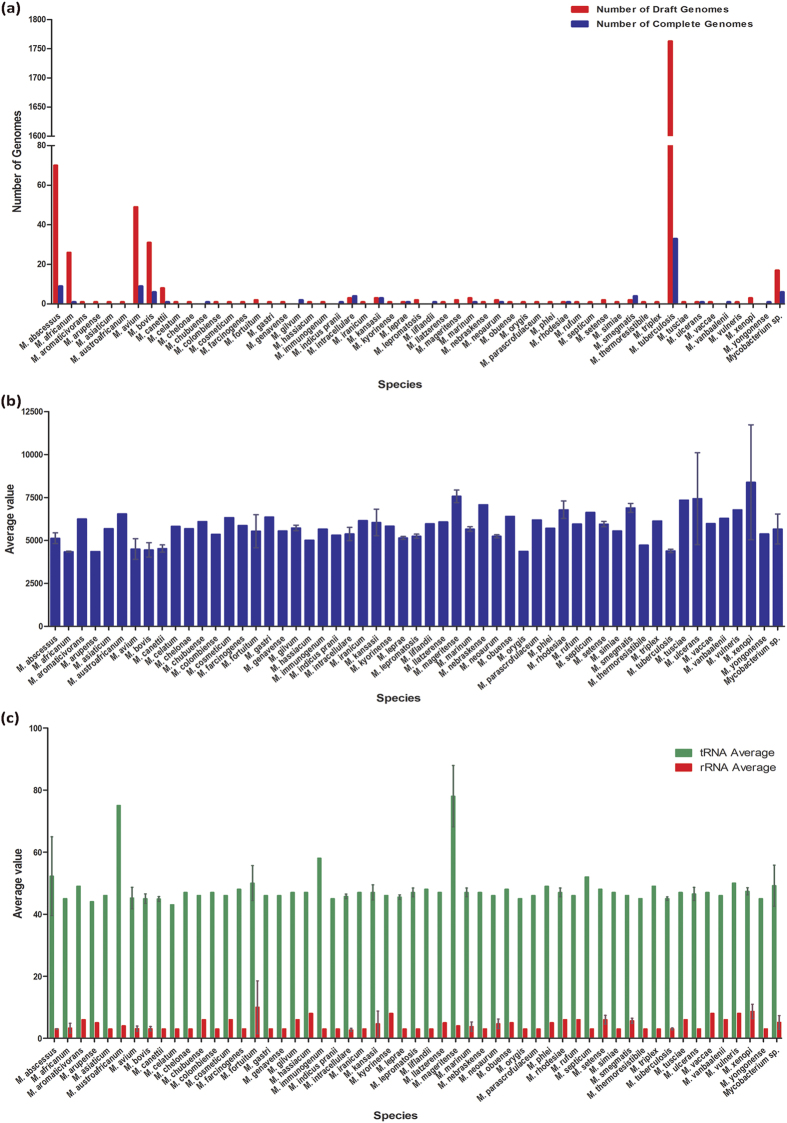
Genome resource of MycoCAP. (**a**) Bar graph showing the number of complete and draft genomes of each mycobacterial species available in MycoCAP. (**b**) Bar graph showing the average number of ORFs in each mycobacterial species available in MycoCAP. (**c**) Bar graph showing the average number of tRNAs and rRNAs present in each mycobacterial species available in MycoCAP.

MycoCAP currently hosts a total of 2108 genome sequences of the *Mycobacterium* genus encompassing at least 55 species (Fig. 2a) with 9,542,758 CDSs (as well as gene annotation), 102,403 RNAs, and 95,834 tRNAs. The highest number of ORFs in a genome was observed in the species *M. xenopi* with an average of ~8383 ORFs per genome whereas the lowest number was observed in *M. africanum* with an average of ~4348 ORFs per genome. The distribution of the ORFs, tRNAs and rRNAs among the 55 species is shown in the Fig. 2b,c.

### Browsing MycoCAP

The homepage of MycoCAP provides a brief description of the genus *Mycobacterium* along with the database summary and manually curated information such as news, blogs, recently published papers related to *Mycobacterium* and links to some external resources in the side panel. From the homepage, user can access the other pages of the database such as Browse, Search, Tools, Genome Browser and Download, the links to which are provided in the top panel.

The “Browse” page of MycoCAP lists all the mycobacterial species currently available in this database. For each species, the numbers of complete and draft genomes are shown along with a “View Strains” link in a tabular form. This link takes the user to the “Browse Strains” page which gives a brief description of the species and lists all the strains of that particular species in the form of a table. Users can obtain strain-specific information such as the genome size, number of coding sequences, number of tRNAs and rRNAs and its GC content (%) along with links to external resources like “Taxon” (linking the strain to the corresponding taxonomic classification page in National Center for Biotechnology Information (NCBI)) and “GOLD” (linking to its page in Genome Online Database (GOLD)). Each strain is also provided with a “Detail” button which will lead the user to the ORF browsing page of the particular strain. This page lists the ORFs of the strain along with the ORF ID, functional classification, Contig ID, start and stop positions. Each ORF ID is linked to its corresponding page in NCBI while the Contig ID of each ORF, is linked to the corresponding contig information available in NCBI. Each ORF is also provided with a “Detail” button which leads the user to the “ORF Detail” page providing detailed information of the ORF, such as its ORF ID and type, function, strand, its start and stop positions, nucleotide and amino acid sequence lengths, Hydrophobicity (pH) and Molecular Weight (Da) along with the nucleotide and amino acid sequences. The user also has the option to visualize the position of the ORF in the genome graphically using the customized genome browser embedded in the “ORF Detail” page. The user can download the annotation details, the nucleotide and the amino acid sequences using the Download option provided in the “ORF Detail” page. Alternatively, users can perform a quick search for information under the database Search tab by applying query filters either strain type, ORF ID or relevant keywords, without having to screen through the full list of available strains.

### Data search in MycoCAP

#### Keyword and text-based searches

To swiftly search a particular gene of interest in MycoCAP, we have implemented a robust and real-time Ajax-based search engine to direct the users straight to the gene of interest. MycoCAP supports two ways to perform a standard text-based query: (1) either through the quick text search provided in the homepage or, (2) a dedicated search option accessible under the “Search” tab. Both methods will result in a browsable list of related genes based on users’ keywords. Quick text search allows users to type in keywords, and a list of suggested genes will be shown in real-time manner. The advanced search has a more fine grain filters based on species, strain, keywords, ORF ID and ORF type. Both query methods utilize asynchronous communications between server and client with the use of Ajax technology, offering seamless interaction for the users while using the search facilities.

#### Sequence searches

Searchers for similar sequences are implemented with the BLAST in the MycoCAP. This foundation tool provides alignment information and findings for proteomics and genomic differences through sequence homology search in MycoCAP. BLAST is an algorithm that was created to compare query sequence with a library of databases of sequence (e.g. nr, nt and many more), and to identify library sequences that resembles the similarity of the submitted query sequence above a certain threshold value^12^,^13^. MycoCAP provides the user with two different BLAST search options. A standard BLAST interface, with the option to perform CDS nucleotide sequences comparisons (BLASTN), *Mycobacterium* whole-genome sequences comparisons (BLASTN whole-genome), protein comparisons (BLASTP), and nucleotide with protein comparisons (BLASTX). The users can also select whether to search against (i) all the *Mycobacterium* genomes, (ii) a single or multiple genomes, or (iii) in the case of nucleotide search, against genomic sequences or protein-coding sequences only and also have the option to set the cut-off for the BLAST expect value and turn on/off a filter for low compositional complexity regions. The other BLAST search option is the VFDB BLAST which allows the user to perform a sequence similarity search against the VFDB database^14^,^15^,^16^. The incorporation of VFDB BLAST into MycoCAP is aimed to facilitate our database users to identify whether their genes of interest are potential virulence genes or not, based on the sequence similarity.

### *Mycobacterium* Genome and Annotation Browser (MGAB)

Another tool implemented in MycoCAP is MGAB, a fast and modern JavaScript-based genome browser that we customized from JBrowse^11^ to enable high speed and smooth visualization of contigs, DNA sequences, RNA sequences and genome annotation results of the user selected *Mycobacterium* genome. MGAB can be used to navigate genome annotations over the web and helps preserve the user’s sense of location by avoiding discontinuous transitions, offering smooth animated panning, zooming, navigation and track selection. The customized MGAB genome browser requires three inputs from the user: the species, the strains and finally the contig which the user wants to visualize. It then utilizes the information of the start and end locations, predicted function and subsystem information to display the strand and provides information on it. MGAB will refresh and reload the web page as soon as user changes any of the inputs.

### Comparative Analysis Tools

#### PathoProT: Online analysis tool for *Mycobacterium* comparative pathogenomic analysis

Pathogenicity which refers to the ability of an organism to cause disease in the host represents a genetic component of the pathogen and the overt damage done to the host is a property of the host-pathogen interactions. Meanwhile, virulence refers to the degree of pathology caused by the organism is usually correlated with the ability of the pathogen to multiply within the host and could be affected by other factors. To identify the virulence genes in the mycobacterial genomes, we developed and customized our in-house designed PathoProT pipeline for the analysis of mycobacterial genomes.

To test the functionality of the PathoProT, we used the type strain of all the mycobacterial species as the subject and checked their virulence profiles using the default parameters (Sequence Identity =50% and Sequence Completeness = 50%), (Supplementary Fig. S1a). The combination of heat map and dendrogram showed a neat overview of the clustered strains which have closely related sets of virulence genes present in each group of clustering. From the neatly organized heat map, we could see that the pathogenomic profile of the strains of interest clearly differentiated the more pathogenic ones from the rest. Many of the virulent genes were found to be conserved across the species (Table 1). This clustering also revealed an interesting observation-the rapidly growing *Mycobacterium* species (*M. rhodesiae, M. cosmeticum, M. mageritense, M. neoaurum, M. austroafricanum, M. vanbaalenii, M. gilvum, M. phlei, M. septicum, M. smegmatis, M. thermoresistibile, M. setense, M. fortuitum, M. vaccae, M. iranicum, M. abscessus, M. chelonae and M. chubuense*) tended to cluster together. This clustering pattern was observed even when the stringency level of the pathogenomic analysis was raised (Sequence Identity to 80% and Sequence Completeness to 80%), (Supplementary Fig. S1b).

#### PGC Tool: Comparative and visualization pipeline of two selected *Mycobacterium* strains

*Mycobacterium* species are widely distributed in humans and many of them are frequently associated with a variety of hazardous diseases, such as tuberculosis and leprosy. However we still do not have a very clear understanding of the mycobacterial biology. By comparing genomic sequences, genes, gene order, and other genomic structural landmarks in the mycobacterial genomes, researchers might be able to get a better insight into the relatedness and the variations among the mycobacteria at the genomic level. With that in mind, we developed and incorporated into the MycoCAP, the PGC tool, which allows users to compare and visualize the relatedness between two genomes of interest.

The PGC tool is a consolidated tool for aligning whole-genome sequences using the NUCmer program of MUMmer package^17^, and Circos^18^ for visualization of pairwise comparison between two cross-strain/species. In the Tool’s page of the MycoCAP, users can select the genomes of two *Mycobacterium* strains of interest and compare them with the PGC tool. PGC also allows the users to upload their own genome sequence and compare with any of the strains in MycoCAP database through our custom submission form.

**Figure 3:**
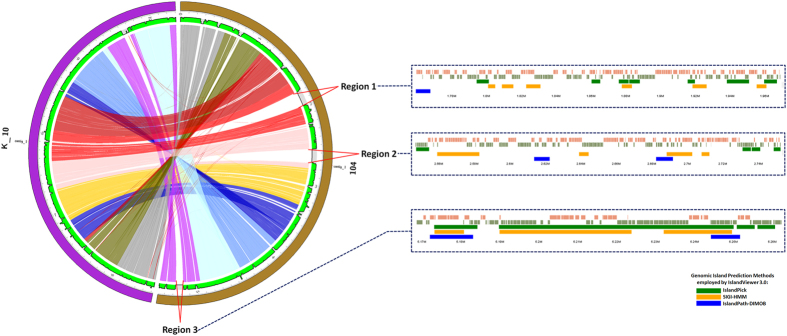
Pairwise genome comparison between *M. avium* 104 and *M. avium* K-10 using PGC tool. (**a**) *M. avium* 104 was used as the reference genome while *M. avium* K-10 was the query genome and the parameters were kept at default. Regions 1, 2 and 3 are the segments in the genome of *M. avium* 104 which are absent in the genome of *M. avium* K-10. The panels shown within the blue dotted box are the genomic islands as predicted by IslandViewer 3.0 correlated with the three gapped regions.

To demonstrate the utility of the PGC, we compared the complete genomes of two strains of *Mycobacterium avium*. We used *M. avium* 104 as the reference genome, while *M. avium* K10 was the query genome and the parameters for the analysis were kept as default. As anticipated, our result revealed that a large portion of the genomes were shared or highly similar since both strains are the same species (Fig. 3). However, clear signs of putative rearrangement events were also observable in certain regions of the genomes. Another interesting observation was the distinct gap regions in the genome of *M. avium* 104, indicating these genomic regions were not shared with *M. avium* K10 genome. To have a clearer view of those gapped regions, we visualized the genome of *M. avium* K10 using MGAB integrated in MycoCAP. Visualization of those regions in higher resolution revealed the presence of mobile elements. Further analysis of those gap regions revealed that they were probable Genomic Island regions as predicted using the IslandViewer 3.0 tool^19^,^20^,^21^ (Fig. 3). We have demonstrated that the PGC tool can be used to investigate and visualize very clearly the genomic differences between two mycobacterial genomes.

#### SuperClassification: *Mycobacterium* Classification Tools

Usually, species identification for rapidly growing mycobacteria is often based on biological and biochemical tests, while antibiotic susceptibility has also been used to assist in species or subspecies classification. But due to events like horizontal gene transfer (HGT) and differential gene expression the accuracy of these phenotypic tests might be affected, as a result of which they are mostly replaced by genotypic method. However a potential drawback of the conventional phylogenetic approach using gene(s) is the optimality of phylogenetic signal in the genes used, due to the lack of a systematic procedure for finding suitable candidates from a population of genes. Studies have shown that 16S rRNA sequence data is not effective in differentiating between all species such as the pathogenic *M. kansasii* from non-pathogenic *M. gastri* and *M. chelonae* from *M. abscessus* due to high levels of 16S rRNA sequence similarity among the *Mycobacterium* species^22^,^23^,^24^. It has been shown that *rpo*B gene of *Mycobacterium* species can clearly separate the slowly growing from the rapidly growing groups of mycobacteria and also effective in differentiating pathogenic *M. kansasii* from non-pathogenic *M. gastri*^25^. It has been reported that *hsp65* gene can be used for the identification of rapidly growing mycobacteria and was also found to be effective in differentiating *M. chelonae* from *M. abscessus*^26^.

To resolve the drawback of the conventional phylogenetic approach, our group has recently implemented and published a systematic relative entropy method to extract optimal phylogenetic information from genes among *M. abscessus*^27^. This systematic or scientific procedure can choose an optimal and informative set of genes for classification of *M. abscessus*. We showed that a minimal set of three genes (*Hoa* coding for 4-hydroxy-2-ketovalerate aldolase, *ftsZ* encoding cell division protein FtsZ and *polC* coding DNA polymerase III subunit alpha) used for phylogenetic tree construction of *M. abscessus* can be as informative as the classical housekeeping genes (*rpoB*, *hsp65*, *secA*, *recA* and *sodA*) for the purpose of inferring a high confidence tree topology.

With that in mind we developed and implemented the SuperClassification tool for the phylogenic classification of the *Mycobacterium* strains based on *rpoB* or *hsp65* as the marker genes and our in-house designed specialized classification system for strains of *M. abscessus*.

Our SuperClassification tool allows user to download the above results upon the completion of their submitted analysis: (1) Radial Tree Image (.svg file), (2) Linear Tree Image (.svg file), (3) Phylogenetic Tree Text (.tre file) and (4) Multiple Sequence Alignment Result (.aligned file). Our tests have shown that if more than 200 genomes are used for the classification, optimum visualization of the classification results are achieved through viewing the downloaded file, instead of online visualization. Hence, the users could provide their Email ID, so that the generated tree can be mailed to the user, which can be downloaded and viewed.

**Figure 4:**
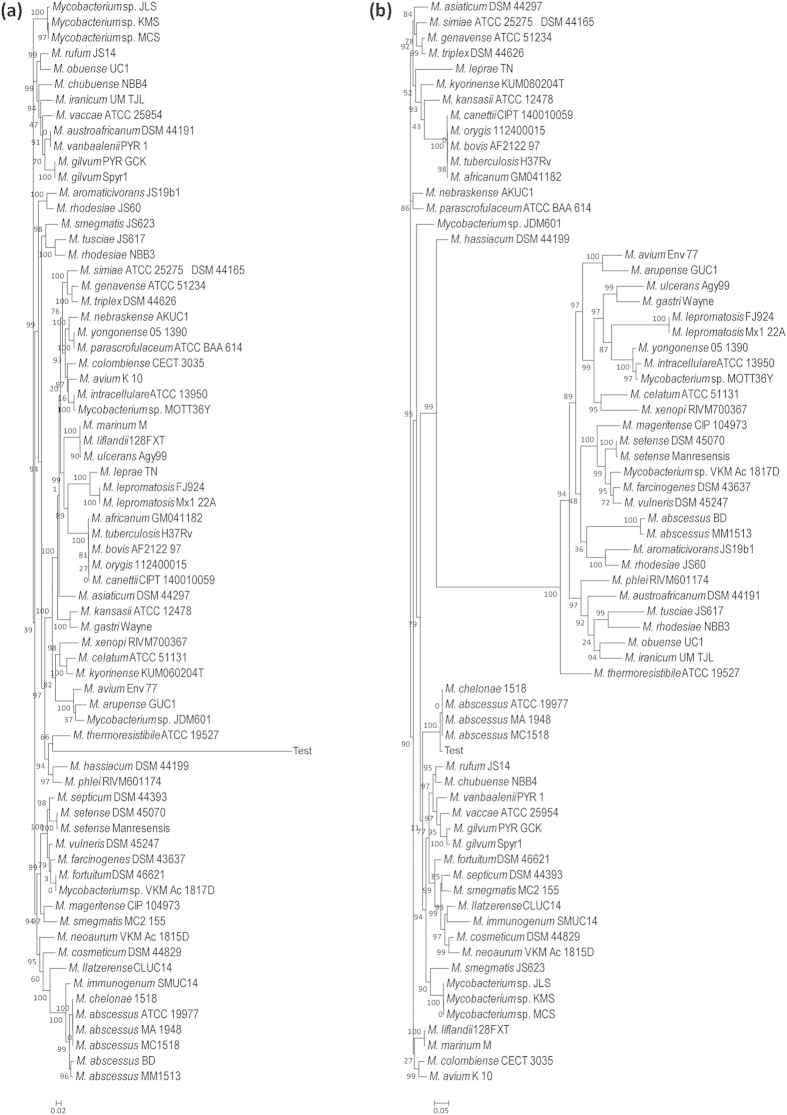
Classification of *Mycobacterium* type strains. (**a**) Classification of *Mycobacterium* type strains using the *rpoB* gene marker. **(b)** Classification of *Mycobacterium* type strains using the *hsp65* gene marker.

**Figure 5:**
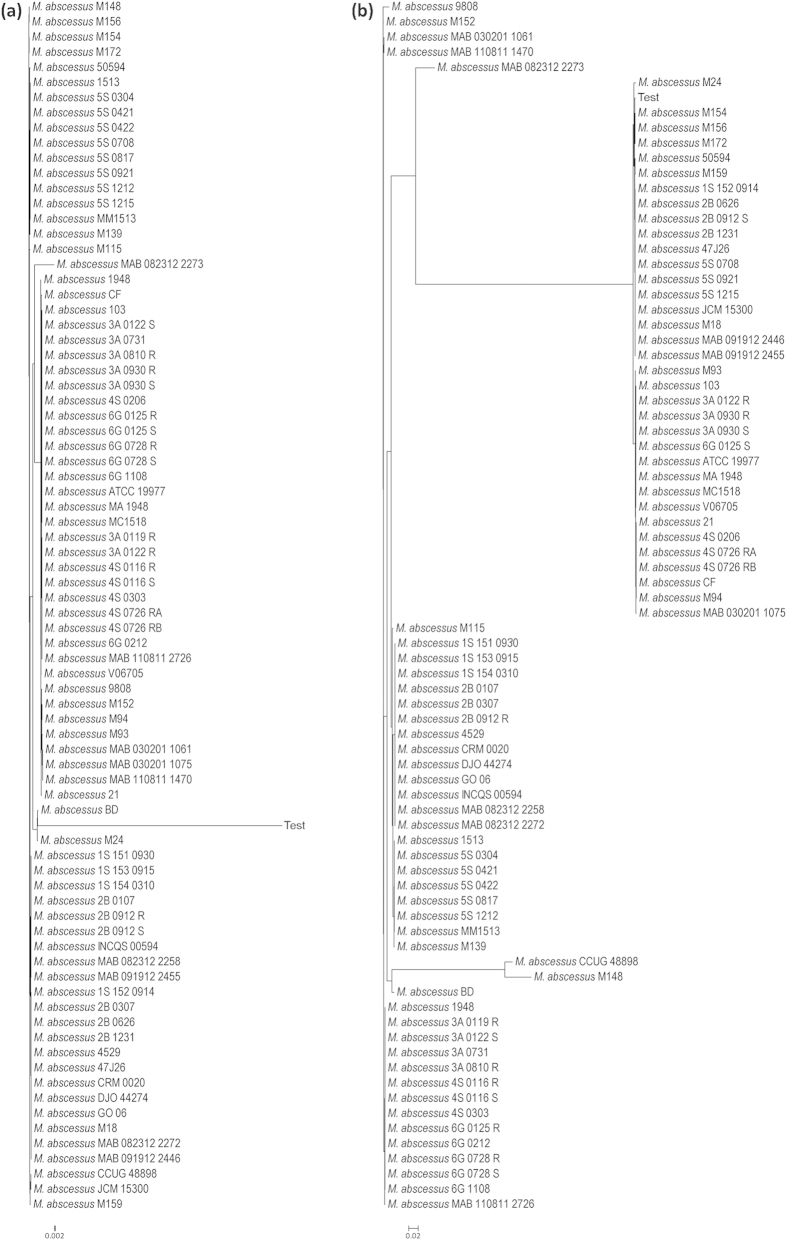
Classification of *M. abscessus*. (**a**) Classification of *M. abscessus* using the *rpoB* gene marker. (**b**) Classification of *M. abscessus* using the *hsp65* gene marker.

To test the classification tool, we first generated two trees using *rpoB* and *hsp65* as the marker genes of all the type strains of all the mycobacterial species. We looked for the classification pattern of the 5 *M. abscessus* strains used for the analysis for both the marker genes. We observed that in case of the *rpoB* marker gene, all the 5 *M. abscessus* strains were clustered together. However they were segregated into two separate branches with *M. abscessus* BD and *M. abscessus* MM1513 clustering together while the other three in a different branch (Fig. 4a). A similar kind clustering pattern was observed in case of *hsp65* marker gene, however this time the two branches were quite apart from each other (Fig. 4b). To investigate further, we generated two new trees using *rpoB* and *hsp65* as the marker genes of all the 79 strains of *M. abscessus* available in MycoCAP. It was interesting to see that in both the trees the three *M. abscessus* strains (ATCC19977, MA1948 and MC1518) remained clustered together however the other two strains (BD and MM1513) separated from each other and clustered in completely different branches (Fig. 5).

**Figure 6:**
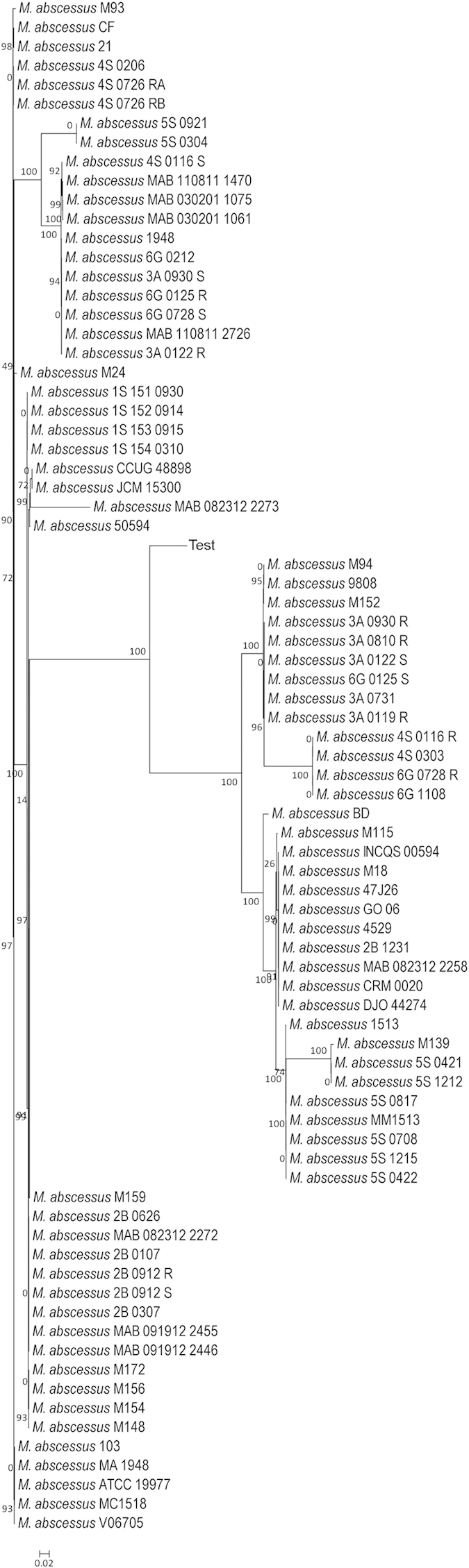
SuperClassification of *M. abscessus*. Classification of *M. abscessus* based on the combined sequence of 4-hydroxy-2-ketovalerate aldolase (*Hoa*), Cell division protein FtsZ (*ftsZ*) and DNA polymerase III subunit alpha (*polC*).

To test the classification tool dedicated to *M. abscessus*, we generated another tree using all 79 strains of *M. abscessus* available in MycoCAP (Fig. 6). As mentioned in the previous section, this tool uses three marker gene sequences of (1) 4-hydroxy-2-ketovalerate aldolase (*Hoa*) (2) Cell division protein FtsZ (*ftsZ*) and (3) DNA polymerase III subunit alpha (*polC*) to classify the *M. abscessus* strains. Our results showed a similar clustering pattern of the five strains with the three *M. abscessus* strains (ATCC19977, MA1948 and MC1518) grouping together in the same branch whereas the other two strains (BD and MM1513) were grouped in completely different branches. The main difference from the previous trees was however the resolution of the clustering pattern might be generally much higher with clearer demarcation between the branches and the strains (Fig. 6).

## Discussion

With advances in the NGS technologies, more and more mycobacterial genomes are being sequenced and analysed. Comparative genomics is expected to play a major role in understanding the evolutionary mechanism of this genus. Here we have presented the design and development of MycoCAP, aiming to be a one-stop genomic resource and comparative analysis platform. A comprehensive overview on the features and analysis tools of MycoCAP was given, which, alongside with selected examples, illustrates the applications and power of MycoCAP as a state-of-the-art comparative analysis web platform for the *Mycobacterium* research community.

In order to provide the most accurate and detailed information about the *Mycobacterium* genus, we will incorporate the new genome sequence releases into MycoCAP as soon as they become available. At present, MycoCAP is optimized for whole-genome analysis. Integration with other data types and genomic resources may make MycoCAP more comprehensive and informative in future. In addition, we also encourage researchers to submit their own information, for example, through e-mail (girg@um.edu.my) or the submission form available at the MycoCAP website which will speed up the improvement of this platform for research community.

## Methods

### Data collection, preprocessing and annotation

All the sequences of 2108 genomes were collected from NCBI (http://www.ncbi.nlm.nih.gov)^28^. For consistency and better comparison, all the *Mycobacterium* genome sequences were re-annotated with the Rapid Annotation using Subsystem Technology (RAST) pipeline^29^. This pipeline is a fully automated annotation engine for complete or draft archaeal and bacterial genomes, capable of identifying various important components in a genome such as protein encoding genes, rRNA and tRNA, pseudogenes, gene functions and subsystems prediction. The pipeline then utilizes this information to construct the metabolic network and generate user-friendly, downloadable results. Protein assignments in the pipeline are based on functional properties, i.e. proteins are predicted according to the closely-relatedness within the subsystems in FIGfams database^30^. All annotations including genes, RNAs and predicted protein functions of *Mycobacterium* strains were stored in the MySQL database.

### MycoCAP Architecture

The web application architecture is a 4-tier web architecture comprising presentation (client), web application (business), and database tiers. The webserver was built on top of Ubuntu Lucid 10.04 by OpenPanel – an open source web server control panel. For the data tier, MySQL 14.12 was used as database. A combination of PHP 5.3 and Perl 5 languages, Codeigniter 2.1.3 framework were employed for development of the web tier, and Twitter Bootstrap front-end framework for presentation layer.

### PathoProT

In the PathoProT, the orthologs of experimentally verified virulence genes of different pathogenic organisms which have been retrieved from the Virulence Factor Database (VFDB)^14^,^15^,^16^, are identified in the user-defined *Mycobacterium* genomes present in the MycoCAP by the use of the well-established BLAST (stand-alone version) tool of NCBI^12^,^13^,^31^,^32^, which was embedded in the pipeline of PathoProT. This pipeline was developed using a combination of in-house Perl and R scripts. The user first needs to select the *Mycobacterium* genomes (from the provided list in the online web form), in which the search for the orthologs of the virulence genes will be made. The default parameters for the BLAST search are set to 50% sequence identity and 50% sequence completeness, however the user can change these default parameters based on their desired levels of stringency. The orthologs thus identified in the *Mycobacterium* genomes are referred to as the “predicted virulence genes”. The in-house developed Perl script handles the early process, where it is programmed to filter the search results generated from BLAST search against VFDB. The selected results are structured into a data matrix form, in which the relationships between strain/genomes and the virulence genes are displayed. The data frame is then reorganized using the Perl script which assigns 1 and 0 values for “presence” and “absence” of virulence genes respectively. The generated data frame will be converted into a data matrix and the initial result is passed on to the R script for hierarchical clustering of the virulence genes. The final output is generated in the form of a heat map, combined with a side-by-side dendrograms giving a bird’s eye view of the distribution of the virulence genes across *Mycobacterium* spp. The combination of heat map and dendrogram shows a neat overview of the clustered strains which have closely related sets of virulence genes present in each group of clustering. In addition, the strains are sorted according to the level of similarities across the strains and virulence genes. The working of the PathoProT pipeline, both how Perl script and R script are integrated, and the processes before generating the output file is demonstrated in Supplementary Fig. S2. To enable the user to further analyze the results generated from PathoProT or use the heat map for publication purposes, the download options for the BLAST alignment result (.txt), overview of virulence genes (.txt) and the Heat Map (.pdf) are provided.

### PGC

In PGC, one of the genomes will be considered as a reference genome while the other one will be labelled as query genome for comparison purposes. Immediately after the alignment files are generated from NUCmer, PGC pipeline will parse the results to Circos to generate a circular ideogram layout showing the relationship between pairs of genomes, with karyotypes and links encoding the position, size and orientation of the related genomic elements. This multi-step process of the pipeline was automated using Perl scripts and the results can usually be visualized in just a few minutes. The results generated by the PGC tool, both the alignment results and the Circos plot alongside with a help file can be downloaded by clicking on the “Download” button in the PGC result page. Supplementary Figure S3 briefly describes the workflow of PGC tool, describing the integration of both MUMmer and Circos and to generate the required input files for the PGC to be function.

PGC allows users to tailor their desired analyses by enabling them to configure parameters such as: (1) minimum percent genome identity (%), (2) link threshold [LT] (bp), which removes the links according to user-defined value and (3) merge threshold [MT] (bp), which allows merging of links based on user-defined value. By default, the thresholds of the PGC tool are set to be 95% minimum percent identity and 1,000 bp link threshold. Users may change the parameters to get different comparative results. The influences of different parameters on the comparative results and the display of the aligned genomes are shown in Supplementary Fig. S4. Additionally, users can input their email addresses for the analysis result to be sent directly to their emails once the analysis is completed by the PGC pipeline.

Development of PGC was inspired by similar tools, such as Circoletto^33^ and RCircos^34^. However, our in-house designed PGC tool has added and distinct advantages over these existing tools. For instance, the alignment algorithm used in PGC is based on the NUCmer package in MUMmer, which is more suitable for large-scale and rapid genome alignment^35^, instead of BLAST (local alignment) as employed by the Circoletto. Apart from the fact that PGC allows the user to tailor their analysis by configuring the parameters, it also provides a histogram track in the circular layout, which shows the percentage of mapped regions along the genomes. This track is very useful in identifying putative indels and repetitive regions in the compared genomes. RCircos, another tool designed with similar function to PGC, was developed using R packages that comes with R base installation^34^. However, PGC has a distinct advantage over RCircos by providing a user-friendly web interface, which unlike RCircos, requires no prior knowledge in programming languages in running the package.

### SuperClassification

In the SuperClassification pipeline, for the classification of all the *Mycobacterium* species the marker gene sequences (*rpoB* or *hsp65* depending on the choice of the user) of the selected genomes are first aligned following which the tree is constructed. While the classification pipeline, specifically for the strains of *M. abscessus*, which is based on 3 different gene sequences: (1) 4-hydroxy-2-ketovalerate aldolase (*Hoa*) (2) Cell division protein FtsZ (*ftsZ*) and (3) DNA polymerase III subunit alpha (*polC*), has an intermediate step before the alignment process. The three sequences are first used to generate a supersequence for each strain, which then undergo the multiple sequence alignment process. The SuperClassification tool uses the MAFFT tool^36^,^37^ for the multiple sequence alignment after which the aligned sequences are then submitted to FastTree2 software^38^,^39^ for phylogenetic tree reconstruction. Supplementary Figure S5 briefly describes the SuperClassification pipeline for MycoCAP.

### Data Download

Users can download all of these genome sequences and annotations through the “Download” page of MycoCAP. Through the provided interactive web forms, users can select which data and annotations to download. Alternatively, users can download these data with a File Transfer Protocol (FTP) download option provided in the “Download” page, which allows the users to batch download the sequence and annotation files (Supplementary Fig. S6).

## Additional Information

**How to cite this article**: Woh Choo, S. *et al.* MycoCAP - *Mycobacterium* Comparative Analysis Platform. *Sci. Rep.* **5**, 18227; doi: 10.1038/srep18227 (2015).

## Supplementary Material

Supplementary Information

## Figures and Tables

**Table 1:** Predicted virulence genes conserved in all the representative strains of 55 mycobacterial species (Sequence Identity = 50% and Sequence Completeness = 80%).

Virulence Genes	Description
glnA1	GLUTAMINE SYNTHETASE GLNA1 (GLUTAMINE SYNTHASE) (GS-I)
leuD*	ISOPROPYLMALATE ISOMERASE SMALL SUBUNIT
lysA*	PROBABLE DIAMINOPIMELATE DECARBOXYLASE lysA (DAP DECARBOXYLASE)
proC	PYRROLINE-5-CARBOXYLATE REDUCTASE
purC	PHOSPHORIBOSYLAMINOIMIDAZOLE-SUCCINOCARBOXAMIDE SYNTHASE
hbhA	IRON-REGULATED HEPARIN BINDING HEMAGGLUTININ hbhA (ADHESIN)
mma4	METHOXY MYCOLIC ACID SYNTHASE 4 mmaa4 (METHYL MYCOLIC ACID SYNTHASE 4) (MMA4) (HYDROXY MYCOLIC ACID SYNTHASE)
cmaA2	CYCLOPROPANE-FATTY-ACYL-PHOSPHOLIPID SYNTHASE 2 cmaA2 (CYCLOPROPANE FATTY ACID SYNTHASE) (CFA SYNTHASE) (CYCLOPROPANE MYCOLIC ACID SYNTHASE 2) (MYCOLIC ACID TRANS-CYCLOPROPANE SYNTHETASE)
pcaA	MYCOLIC ACID SYNTHASE pcaA (CYCLOPROPANE SYNTHASE)
kasB	3-OXOACYL-(ACYL CARRIER PROTEIN) SYNTHASE
panC	PANTOATE–BETA-ALANINE LIGASE
ideR	IRON-DEPENDENT REPRESSOR AND ACTIVATOR ideR
relA	PROBABLE GTP PYROPHOSPHOKINASE relA (ATP:GTP 3′-PYROPHOSPHOTRANSFERASE) (PPGPP SYNTHETASE I) ((P)PPGPP SYNTHETASE) (GTP DIPHOSPHOKINASE)
mprA	MYCOBACTERIAL PERSISTENCE REGULATOR mrpA (TWO COMPONENT RESPONSE TRANSCRIPTIONAL REGULATORY PROTEIN)
mprB	PROBABLE TWO COMPONENT SENSOR KINASE mprB
prrA*	TWO COMPONENT RESPONSE TRANSCRIPTIONAL REGULATORY PROTEIN prrA
sigA/rpoV	RNA POLYMERASE SIGMA FACTOR
sigE	RNA POLYMERASE SIGMA-70 FACTOR
whiB3	TRANSCRIPTIONAL REGULATORY PROTEIN WHIB-LIKE whib3
fbpA	MAJOR FERRIC IRON BINDING PROTEIN
fbpB	IRON-UPTAKE PERMEASE INNER MEMBRANE PROTEIN
fbpC	IRON-UPTAKE PERMEASE ATP-BINDING PROTEIN
pknG	SERINE/THREONINE-PROTEIN KINASE pknG (PROTEIN KINASE G) (STPK G)
secA2*	TRANSLOCASE
sodC	PROBABLE PERIPLASMIC SUPEROXIDE DISMUTASE [CU-ZN] sodC

^*^Conserved at both 50% and 80% of Sequence Identity and Sequence Completeness.
