# Supplementary material for: MycoCAP - Mycobacterium Comparative Analysis Platform
Source: Sci Rep. 2015 Dec 15;5:18227. doi: 10.1038/srep18227 (PMC4678330; doi:10.1038/srep18227)

MycoCAP - *Mycobacterium* Comparative Analysis Platform

Siew Woh Choo[1,2*], Mia Yang Ang[1,2], Avirup Dutta[1*], Shi Yang Tan[1,2], Cheuk Chuen Siow[1], Hamed Heydari[1,3], Naresh V.R. Mutha[1], Wei Yee Wee[1,2] and Guat Jah Wong[1,2]

[1]Genome Informatics Research Laboratory, High Impact Research Building, University of Malaya, 50603 Kuala Lumpur, Malaysia

[2]Department of Oral Biology and Biomedical Sciences, Faculty of Dentistry, University of Malaya, 50603 Kuala Lumpur, Malaysia

[3]Computer Science and Engineering Department, University of Nebraska-Lincoln, Lincoln, NE, 1468588-0115 USA.

* =Corresponding authors:

Siew Woh Choo (lchoo@um.edu.my)

Avirup Dutta (avirupdutta@gmail.com)

Legends of Supplementary Figures

Supplementary Figure S1: PathoProT generated a heat map showing the clustering of the virulence genes in the type strains of *Mycobacterium*. (a) Heat map generated using the default parameters of Sequence Identity to 50% and Sequence Completeness to 50%. **(b)** Heat map generated using the parameters of Sequence Identity to 80% and Sequence Completeness to 80%.

Supplementary Figure S2: Flow chart briefly describing the PathoProT workflow, from user input sequence until how the result is generated.

Supplementary Figure S3: Flow chart showing the steps taken in PGC pipeline.

Supplementary Figure S4: Influence of different parameters of PGC on the final result. Diagram showing the influence of different parameters of Minimum Percent Identity (%), Link Threshold (LT) and Merge Threshold (MT) on the final output of PGC.

Supplementary Figure S5: Flow chart describing the SuperClassification pipeline of MycoCAP. This pipeline allows users to classify either *Mycobacterium* strains or *M. abscessus* strain of their own with the *Mycobacterium* strains deposited in MycoCAP.

Supplementary Figure S6: Download options in MycoCAP. Users can download *Mycobacterium* genomics data and annotations through our online web form or ftp (for batch download).

Supplementary Figure S1(a)

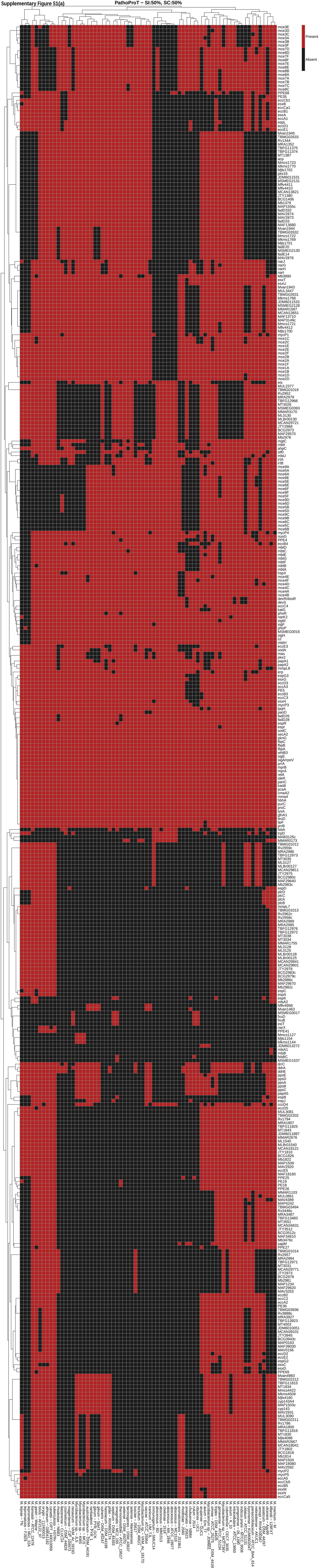

Supplementary Figure S1(b)

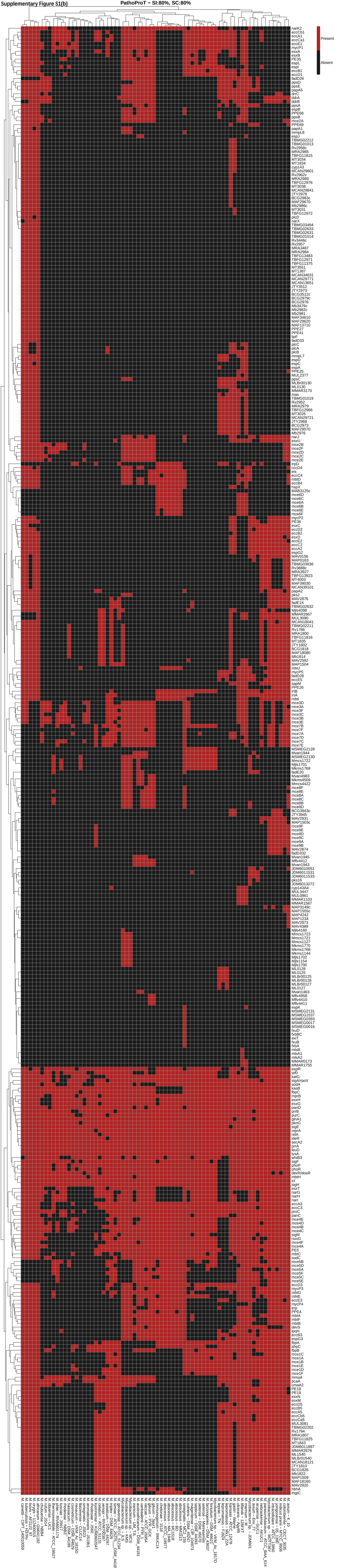

Supplementary Figure S2

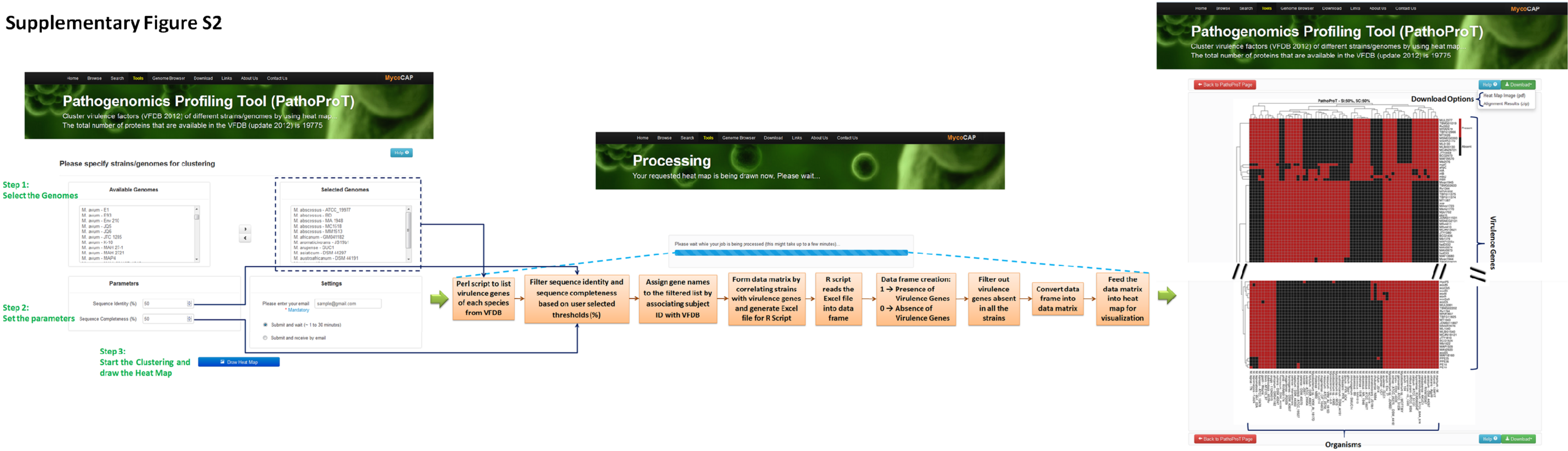

Supplementary Figure S3

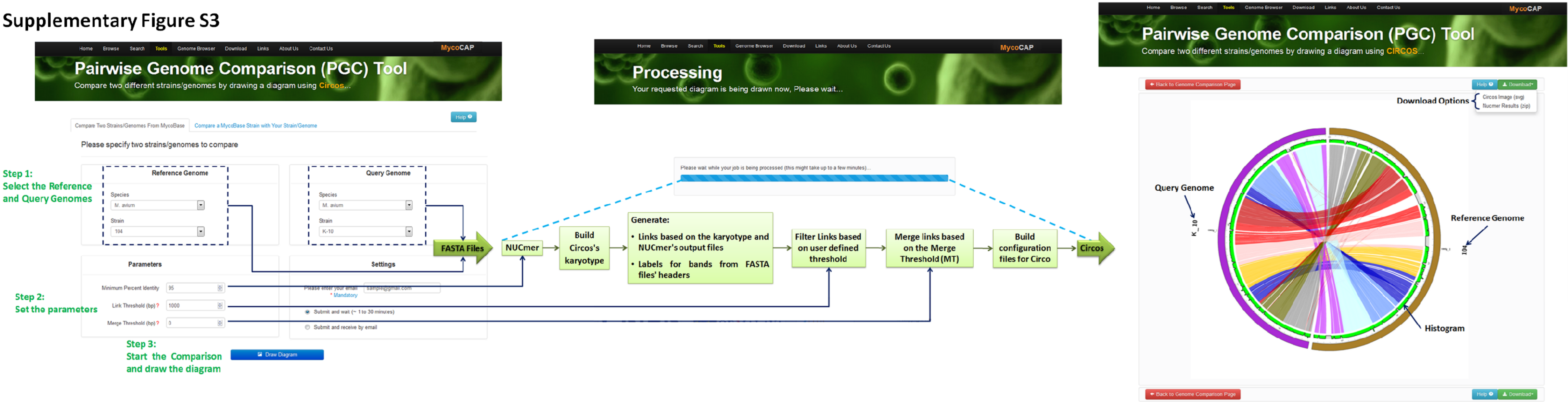

Supplementary Figure S4

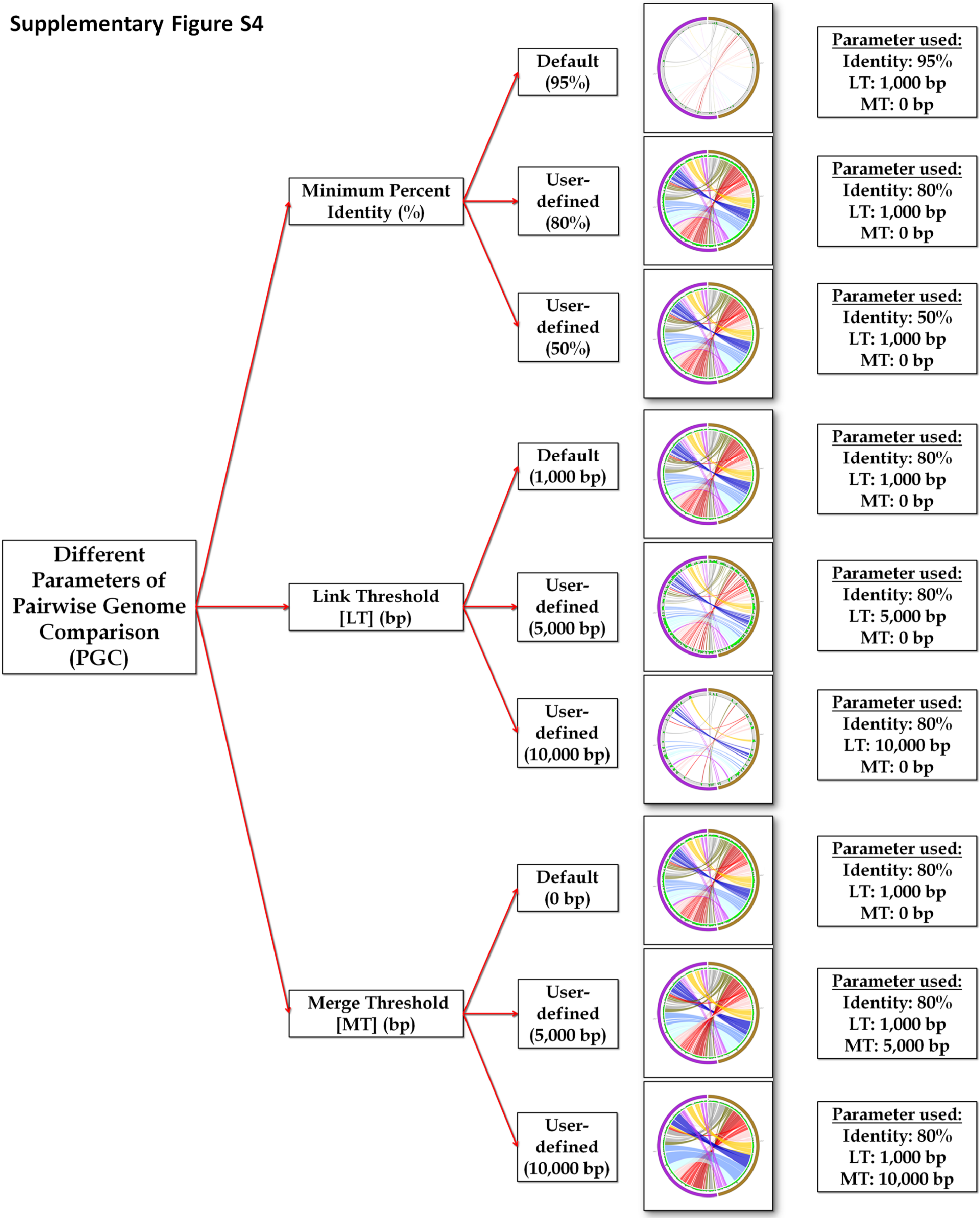

Supplementary Figure S5

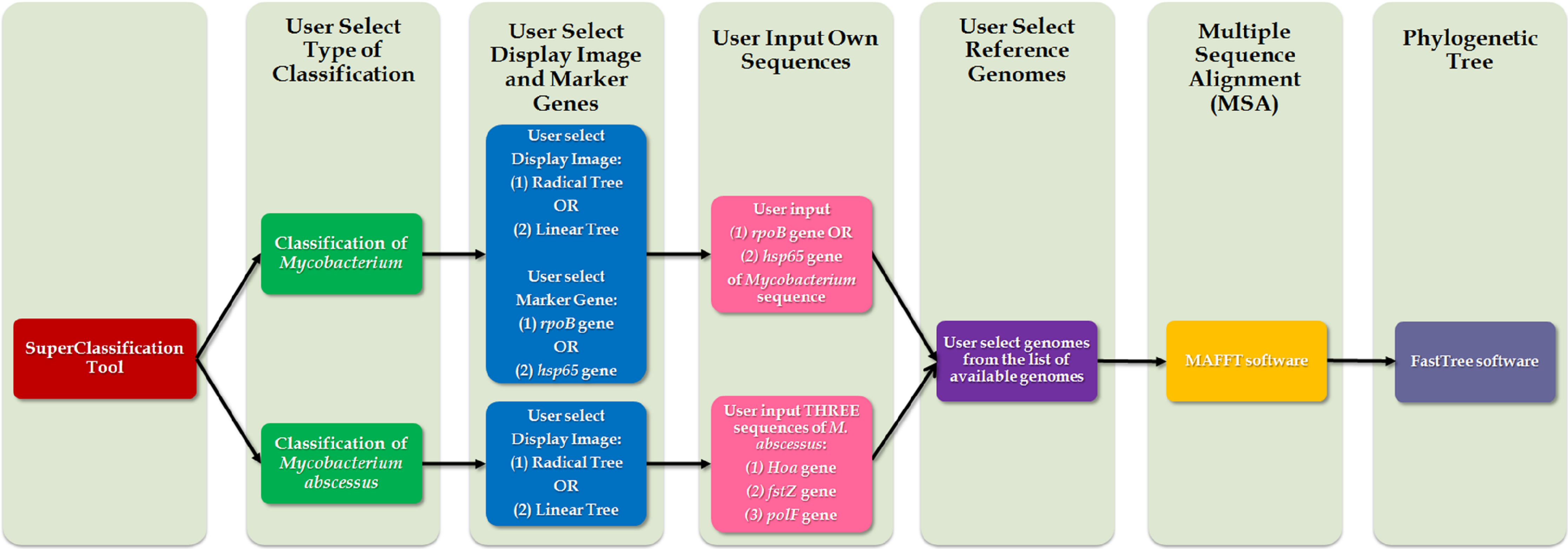

Supplementary Figure S6

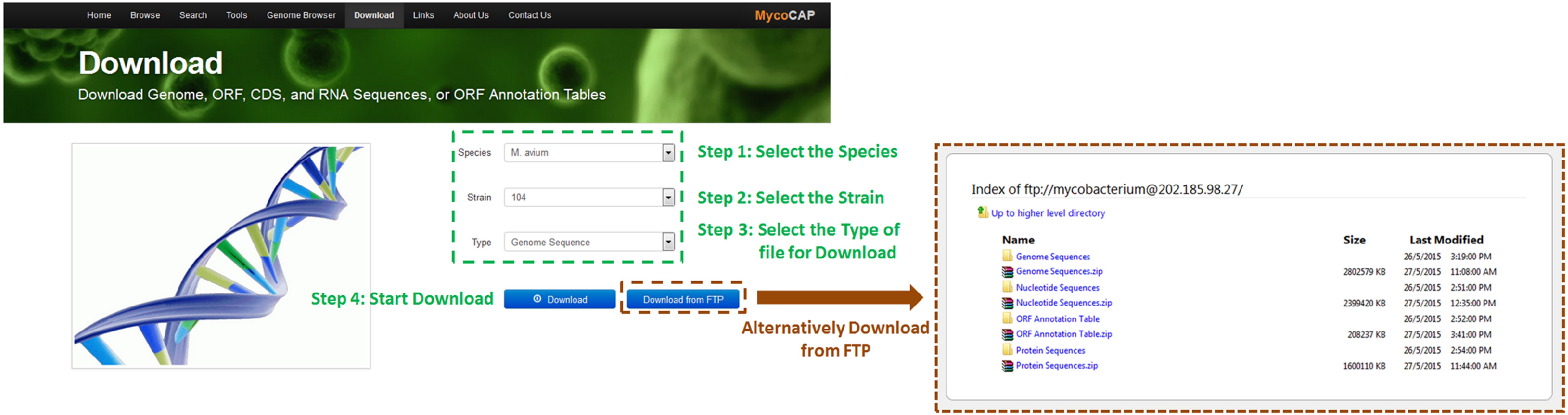

Supplement: Supplementary Information [file srep18227-s1.pdf]
